# Professor Parker

**Published:** 1836

**Authors:** 


### Art. III.—Professor Parker.

It will be seen by the advertisement of the Faculty of the Cincinnati College that this eloquent lecturer, and able surgeon has accepted a chair in that Institution. Few men have arisen as rapidly in the profession as professor Parker. As an orator and teacher of surgery he has few if any superiors in any country. He already holds several appointments in the schools of New England and New York, most or all of which he proposes to resign and emigrate to our city, in order to unite with the Faculty of the Cincinnati school, which is rising rapidly in the favor of the profession both at home and abroad. W. W.

